# MC3T3-E1 Cells on Titanium Surfaces with Nanometer Smoothness and Fibronectin Immobilization

**DOI:** 10.1155/2012/743465

**Published:** 2012-05-22

**Authors:** Tohru Hayakawa, Eiji Yoshida, Yoshitaka Yoshimura, Motohiro Uo, Masao Yoshinari

**Affiliations:** ^1^Department of Dental Engineering, Tsurumi University School of Dental Medicine, 2-1-3 Tsurumi, Tsurumi-ku, Yokohama 230-8501, Japan; ^2^Department of Molecular Cell Pharmacology, Hokkaido University Graduate School of Dental Medicine, Kita 13, Nishi 7, Kita-ku, Sapporo 060-8586, Japan; ^3^Advanced Biomaterials, Division of Oral Health Sciences, Department of Restorative Sciences, Graduate School of Medical and Dental Sciences, Tokyo Medical and Dental University, 1-5-45, Yushima, Bunkyo-ku, Tokyo 113-8549, Japan; ^4^Division of Oral Implants Research, Oral Health Science Center, Tokyo Dental College, 1-2-2 Masago, Mihama-ku, Chiba 261-8502, Japan

## Abstract

The present study was aimed to evaluate the viability and total protein contents of osteoblast-like cells on the titanium surface with different surface mechanical treatment, namely, nanometer smoothing (Ra: approximately 2.0 nm) and sandblasting (Ra: approximately 1.0 **μ**m), and biochemical treatment, namely, with or without fibronectin immobilization. Fibronectin could be easily immobilized by tresyl chloride-activation technique. MC3T3-E1 cells were seeded on the different titanium surfaces. Cell viability was determined by MTT assay. At 1 day of cell culture, there were no significant differences in cell viability among four different titanium surfaces. At 11 days, sandblasted titanium surface with fibronectin immobilization showed the significantly highest cell viability than other titanium surface. No significant differences existed for total protein contents among four different titanium surfaces at 11 days of cell culture. Scanning electron microscopy observation revealed that smoothness of titanium surface produced more spread cell morphologies, but that fibronectin immobilization did not cause any changes of the morphologies of attached cells. Fibronectin immobilization provided greater amount of the number of attached cells and better arrangement of attached cells. In conclusion, the combination of sandblasting and fibronectin immobilization enhanced the cell viability and fibronectin immobilization providing better arrangements of attached cells.

## 1. Introduction

 Various mechanical and chemical surface modifications of titanium dental implants, such as blasting, alkaline treatment, or hydroxyapatite coating, have been reported for improving the bone response during healing process [1–4]. Some reported the effectiveness of the surface coating or immobilization with cell adhesive protein, such as, fibronectin (FN) or laminin, for enhancing cell attachment, cell spreading, and cell activity [5, 6].

**Figure 1:**
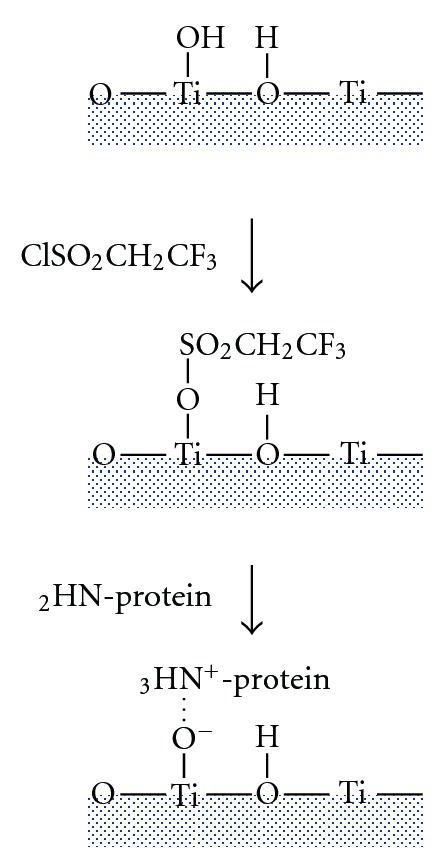
Presumed mechanism for fibronectin immobilization using tresyl chloride-activated technique.

For biochemical surface treatment, several methods for covalent immobilization of cell adhesive proteins have been reported. Silane coupling reagents have been widely used for the immobilization of cell adhesive proteins [7–10]. On the contrary, Hayakawa et al. reported an easy and simple method for the immobilization of cell adhesive proteins onto a titanium surface, which was named the tresyl chloride-activated method [11, 12]. This method was a modified version of the method reported by Nilsson and Mosbach [13]. Tresyl chloride (2,2,2-trifluoroethanesulfonyl chloride) is a liquid. Hayakawa et al. applied tresyl chloride directly on the titanium surface without the use of any solvent and found the basic hydroxyl groups of the titanium surface reacted with tresyl chloride [11, 12]. Cell adhesive protein could be easily immobilized onto titanium surface through ionic interaction as shown in Figure 1, which was confirmed by quarts-crystal microbalance-dissipation measurements [14]. Fibronectin-, collagen-, and fibronectin-derived peptides could be easily immobilized onto a titanium surface. Gene expression of osteoblast-like cells was monitored on titanium immobilized with fibronectin or fibronectin-derived GRGDSP peptide and it was found that the expression levels of some genes related to the mineralization process, for example, bone sialoprotein and osteomodulin, were upregulated [15–17].

 Roughness of a titanium surface also influenced the bone response after the implantation. Many studies have shown that a rougher surface provided better bone formation or osteoconduction compared with a smooth surface [18]. However, the issues of optimal surface roughness and superficial morphology are still controversial and need to be clarified. Larsson et al. reported that differences of surface roughness between Ra = 30.3 **μ**m (machined) and Ra = 2.9 nm (electropolishing) varied the amount of bone after 6 weeks of implantation in rabbit cortical bone but not after 1 year of implantation [19]. Wu et al. investigated human fetal osteoblastic cell behavior on titanium surfaces with different roughness [20]. They reported that the roughest plasma-sprayed surface (Ra = 33 nm) exhibited the highest number of cell attachment cells and ALP activity. However, they also reported that the ALP level of the polished surface with nanometer smoothness (Ra = 6 nm) was much higher than that of the satin- (Ra = 0.83 *μ*m) and grit-blasted (Ra = 11 *μ*m) groups at 16 days of cell culture.

 Previously we investigated the influence of two different surface roughness, namely, nanometer smoothing (Ra: approximately 2.0 nm) and sandblasting (Ra: approximately 1.0 *μ*m), as well as biochemical treatment, namely, fibronectin immobilization using tresyl chloride-activated technique, of a titanium surface on osteoblast-like cell behavior [21]. It was reported that the nanometer-smooth surface was beneficial for the differentiation of MC3T3-E1 cells and that FN immobilization provided better arrangement of attached cells.

In the present study, we aimed to evaluate cell viability of MC3T3-E1 and total protein content on the above-mentioned four different titanium surfaces, as a next series of our experiments. Cell viability was determined by MTT assay. The morphologies of attached cell were observed by scanning electron microscope (SEM).

## 2. Materials and Methods

### 2.1. Nanometer-Smoothing and Sandblasting of Titanium Surface

 Commercially pure titanium disk (*ϕ* = 15 mm × 1.0 mm, JIS, Japan Industrial Specification H 4600, 99.9 mass% Ti, Furuuchi Chemical Corp., Tokyo) was used. Nanometer-smooth titanium surface (Ti-smooth) was prepared by polishing with diamond slurry with diamond particle diameters of 6 *μ*m, 3 *μ*m, and finally 1 *μ*m using a rapping machine (Rapping machine 12 in, TDC, Corp., Miyagi, Japan). After polishing, the specimen was cleaned with ethanol. Sandblasted surface (Ti sand) was prepared by sandblasting with alumina powder (50 *μ*m) at 5 atmospheric pressures for 5 s. After sandblasting, the specimen was cleaned ultrasonically with acetone-distilled water mixture and was then cleaned with 10 wt% HF and 5 wt% HNO_3_ aqueous mixture for 20 s to remove any remaining alumina powder. Finally, the sandblasted specimen was cleaned ultrasonically again with acetone-distilled water mixture.

The surface roughness of nanometer-smooth titanium disk, which was determined with Talysurf CCI3000 (0.35 mm length and 12 nm pitch, Ametek Co., Ltd. Tokyo, Japan), was 2.0 ± 0.9 nm, and that of sandblasted titanium disk determined with SURFCOM-30A (Tokyo Seimitsu, Japan, 4 mm scale length and 0.8 mm pt.) was 1.0 ± 0.7 *μ*m. Five specimens of each type were measured.

### 2.2. FN Immobilization

 FN immobilization on the titanium was performed in accordance with previous reports [11, 12] Ti smooth and Ti sand disks were completely covered with tresyl chloride (Fluka, Buchs, Switzerland) and then stored at 37°C for two days. Tresyl chloride is a liquid with a boiling point of 140-141°C. Afterwards, tresylated titanium disks were washed with double-distilled water followed by double-distilled water-acetone solution (50 : 50) and then dried and stored in a desiccator.

Human plasma fibronectin (Harbor Bio-Products, MA, USA) was dissolved in phosphate-buffered saline (PBS) solution (pH 7.4) at a concentration of 100 *μ*g/mL for cell attachment assay. Tresylated titanium disks were immersed in the fibronectin-PBS solution for 24 hours at 37°C and then rinsed with double-distilled water. Finally, the titanium disks were dried with a gentle stream of dry air and stored in a desiccator. Thus, fibronectin-immobilized Ti smooth (Ti smooth/FN) and Ti sand (Ti-sand/FN) disks were prepared. The FN immobilization was confirmed by an X-ray photoelectron spectroscope (XPS; Axis Ultra, Kratos Analytical, UK), which was equipped with a monochromatized AlK*α* X-ray source operated at 15 kV and 15 mA. The binding energy scale for each spectrum was calibrated against the C1s peak at 284.8 eV. An N1s peak of amide bonds in fibronectin was detected at 399.9 eV.

### 2.3. Cell Culture

 Murine osteoblastic MC3T3-E1 cells (Riken Cell Bank no. RCB1126, Ibaraki, Japan) were used. The cells were cultured in *α*·MEM (Invitrogen, MD, USA) supplemented with 10% heat-inactivated fetal bovine serum, 66.7 *μ*g/mL kanamycin sulfate, and 284 *μ*M L-ascorbic acid 2-phosphate at 37°C in a humidified atmosphere of 95% air and 5% CO_2_. Subsequently, the cells were cultured to subconfluence in 100 mm standard dishes (Falcon, Becton-Dickinson Labware, Franklin Lakes, NJ, USA) and transferred to Bioflex collagen I-coated 24-well plates (Falcon ) after treatment with 0.25% trypsin/EDTA (Invitrogen, MD, USA). The culture medium was changed every three days. Classical medium without any bone differentiation components was employed in the preset study for evaluating the influence of fibronectin immobilization and surface roughness on the cell behavior [22, 23].

Four different surfaces, that are, Ti smooth, Ti smooth/FN,Ti sand, and Ti sand/FN were evaluated by cell assay. Each titanium disk was sterilized with ethyleneoxide gas.

### 2.4. MTT Assay

 MC3T3-E1 cells were seeded on each titanium disc in 24-well plates at a density of 5 × 10^4^ cells/cm^2^ and incubated for 11 days. The culture medium was changed every three days. Cell viability was determined by (3-(4,5-di-methylthiazol-2-yl)-2,5-diphenyltetrazolium bromide (MTT) assay by using ViaLight Plus Kit (Lonza Group Ltd, Basel, Switzerland). At predetermined time intervals (1 and 11 days), attached cells were rinsed with PBS and 200 *μ*L of culture medium was added. Then, 200 *μ*L of the cell lysis reagent was added, and cells were incubated in the orbital shaker for 10 min at 600 rpm, and 100 *μ*L of cell medium was transferred to a white plate. Afterwards, 100 *μ*L of ATP Monitoring Reagent Plus was added, and the plate was incubated for 2 min in the dark condition (inside the luminometer) before the measurement. Measurement was taken with the plate reader (Wallac 1420 ARVOsx, PerkinElmer, Inc., MA, USA). Four runs were performed and four specimens were analyzed for each substrate and each period.

### 2.5. Total Protein Content

 MC3T3-E1 cells were seeded on each titanium disc in 24-well plates at a density of 5 × 10^4^ cells/cm^2^ and incubated for 11 days. To determine the total cellar protein, BCA Protein Assay kit (Thermo Fisher Scientific K.K. MA, USA) was used. 200 *μ*L of the cell lysis reagent was added to each well, and then 10 *μ*L of each specimen was pipette and 90 *μ*L of cell lysis buffer was added into a 96-well plate. 100 *μ*L of the reagent (micro BCA kit; Thermo Fisher Scientific Inc., MA, USA) was added to each well and was mixed. The enzyme reactions were conducted for 30 min at 37°C, and absorbency at 570 nm was immediately measured using a plate reader (Bio Rad model 550, Bio-Rad Laboratories, Inc. CA, USA). Four runs were performed and four specimens were analyzed for each substrate.

### 2.6. SEM Observation

 After 1 day of cell culture, titanium disks were rinsed thoroughly in PBS, mounted on a piece of cork, and fixed in 2.5% glutaraldehyde in phosphate buffer (pH 7.4). Following fixation, disks were dehydrated through a graded series of ethanol (50, 60, 70, 80, 90, 95, and 100%) and then dried with tetramethylsilane. After ion coating with gold, the morphology of the cells was observed using a scanning electron microscope (S4000, HITACHI, Tokyo, Japan) at an accelerating voltage of 5 kV.

### 2.7. Statistics

 Significant differences were determined by one-way analysis of variance (ANOVA) using GraphPad software (Graphpad Prism, GraphPad Software Inc., San Diego, California, USA). Statistical significance was set at *P* < 0.05.

## 3. Results

**Figure 2:**
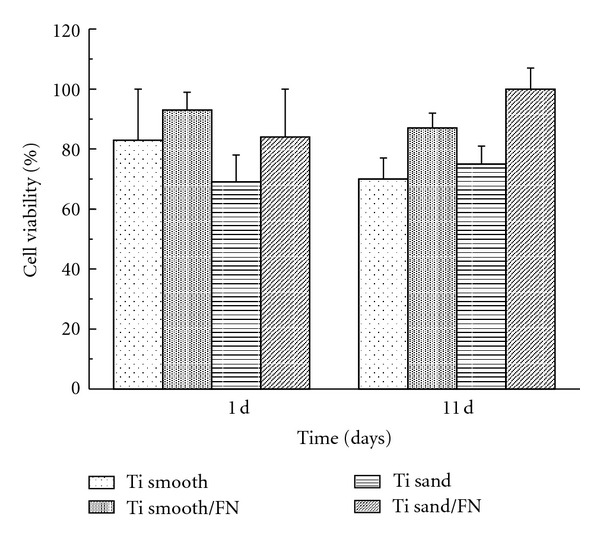
Cell viability determined by MTT assay.

The results of MTT assay are shown in Figure 2. At 1 day of cell culture, there were no significant differences among four different titanium surfaces. At 11 days, Ti sand/FN showed the significantly highest cell viability than Ti sand, no significant differences were detected between Ti smooth/FN and Ti smooth. Between 1 day and 11 days of cell culture, there were no significant differences for each titanium surface.

**Figure 3:**
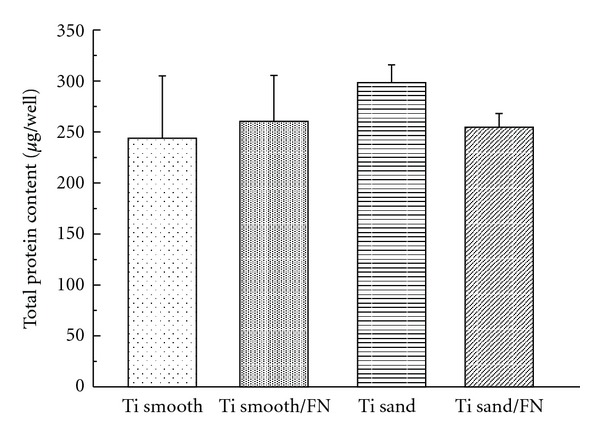
Total protein contents.

Figure 3 shows the results of measurement of total protein contents at 11 days. No significant differences existed for total protein contents among four different titanium surfaces.

**Figure 4:**
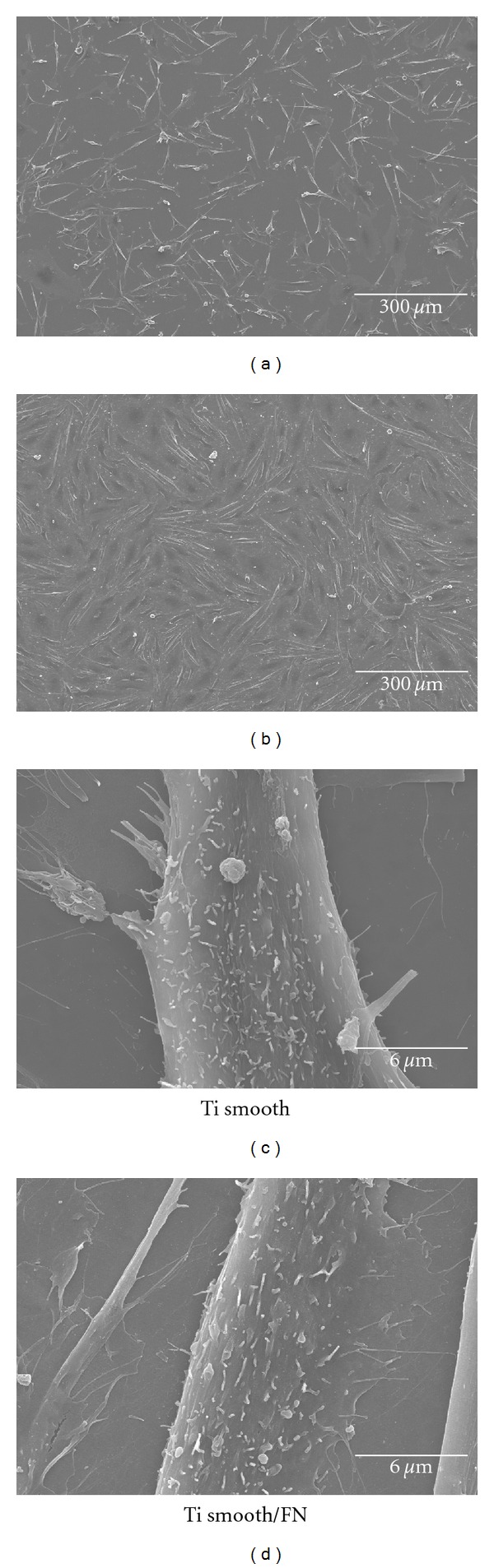
SEM views of MC3T3-E1 cells cultured on Ti smooth and Ti smooth/FN titanium disk at 1 day of culture.

**Figure 5:**
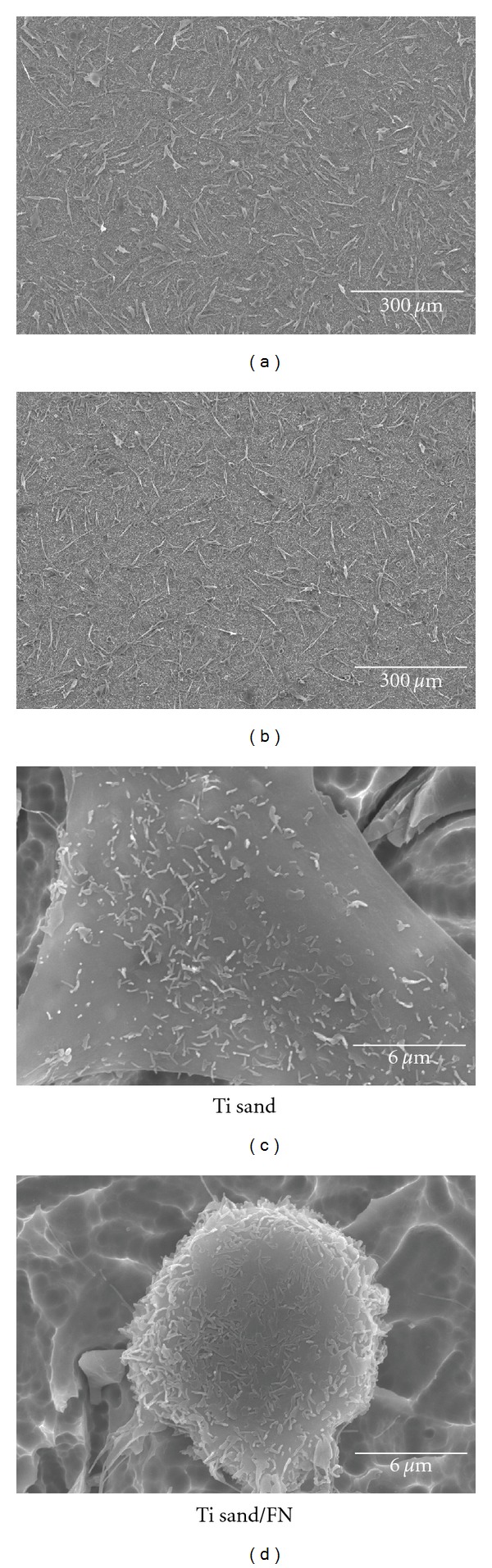
SEM views of MC3T3-E1 cells cultured on Ti sand and Ti sand/FN titanium disk at 1 day of culture.

 Figures 4 and 5 show the SEM views of attached cells after 1 day of cell culture. Smoothness of titanium surface affected the cell morphologies. On the other hand, FN immobilization did not have any influence on the morphologies of attached cells but did have some influence on the number of attached cells.

 Cells attached on nanometer smooth titanium (Ti smooth, Ti smooth/FN) showed the flat shape with a large and thin cytoplasmic layer and with numerous filopodia. The filopodia was extending from the cell body to the titanium surface and higher magnification indicated the presence of short-fiber-like structure, which is presumed to be microvilli. Comparing Ti smooth and Ti smooth/FN, Ti smooth/FN surface showed more attached cells and attached cells showed better arrangement on Ti smooth/FN surface rather than on Ti smooth (Figure 3).

 Attached cells on sandblasted surface (Ti sand and Ti sand/FN) were slightly less spread than on nanometer smooth surface (Ti smooth, Ti smooth/FN). Some of them had a globular appearance, especially cell on Ti sand/FN. More globular structure was observed on Ti sand/FN and higher magnification clearly showed the globular structure of attached cell (Figure 4).

## 4. Discussion

In this study, we evaluated cell viability and total protein contents of MC3T3-E1 cells on four different titanium surfaces, Ti smooth, Ti smooth/FN, Ti sand, and Ti sand/FN. The influences of mechanical treatment (nanometer smoothing and sandblasting) and biochemical treatment (with and without fibronectin immobilization) of a titanium surface were investigated.

 Previously, we monitored DNA amounts, ALP activity, octeocalcin production and mineralization behavior to four different surfaces same as the present study [21]. It revealed that surface roughness enhanced the differentiation of MC3T3-E1 but not FN immobilization.

 The cell viability for Ti sand/FN showed greater cell viability than that for Ti sand. Although the reason is not clear, the combination of sandblasting and FN immobilization provided better cell viability. Only FN immobilization did not affect cell viability.

 Pugdee et al. reported that significantly more MC3T3-E1 cells attached to FN-immobilized titanium disk than to untreated titanium disk at 30 min after cell seeding [16]. However, the present study showed no remarkable enhancement of cell attachment by FN immobilization, which was determined by total protein contents. It is presumed that the controversial results were caused by the difference of cell assay time, 30 min versus 11 days and the difference of number of seeded cells, 1 × 10^5^ versus 5 × 10^4^ cells/cm^2^. FN is a major extracellular matrix protein, and cultured cells produce FN by themselves during cell assay. It concluded that FN immobilization only enhanced the initial cell attachment, not proliferation.

 Cell on Ti sand/FN showed the highest cell viability, but not the highest total protein contents. It is suggested that the activities and/or morphologies of cells on Ti sand/FN were influenced by the combination of surface roughness and FN immobilization. The details of the mechanism are indistinct.

 SEM observation appeared that more attached cells were present on the Ti smooth/FN surface than on Ti smooth. Moreover, well-arranged cells could be observed on Ti smooth/FN. This was corresponded with the previous results [21]. It is presumed that cells can recognize immobilized FN on titanium surface. Therefore, the arrangement of attached cells is controlled by the presence of immobilized FN.

 Mineralization induction medium was not used in the present cell assay experiments. Classical medium without any bone differentiation components was employed in the preset study for evaluating the influence of fibronectin immobilization and surface roughness on the cell behavior [22, 23].

 FN could be easily immobilized on both nanometersmooth and sandblasted titanium surfaces by our originally developed method, namely, the tresyl chloride-activation technique [11, 12]. The advantage of the tresyl chloride-activation technique is its simplicity. The titanium surface needs no pretreatment, such as passive oxidation, alkaline treatment. For example, Nanci et al. immobilized alkaline phosphatase or albumin to titanium [8]. First they treated titanium surface with a mixture of sulfuric acid and hydrogen peroxide to reproduce titanium oxide surface layer and then applied-silane-coupling agents to oxide titanium layer for the immobilization of proteins. Another advantage of tresyl chloride-activation technique is that any types of proteins and cytokine such as BMP or TGF-*β* can be immobilized on titanium. The influence of the immobilization of other types of proteins or cytokines, for example, laminin or BMP, on the cell behaviors should be further investigated.
